# Pseudomyocardial Infarction in a Patient with Severe Diabetic Ketoacidosis and Mild Hyperkalemia

**DOI:** 10.1155/2019/4063670

**Published:** 2019-03-31

**Authors:** Edgar Francisco Carrizales-Sepúlveda, Ángel Noé del Cueto-Aguilera, Raúl Alberto Jiménez-Castillo, Olga Norali de la Cruz-Mata, Mariana Fikir-Ordoñez, Raymundo Vera-Pineda, Dalí Alejandro Hernández-Guajardo, Alejandro Ordaz-Farías, Ramiro Flores-Ramírez

**Affiliations:** ^1^Internal Medicine Department, Hospital Universitario, Universidad Autónoma de Nuevo León, Monterrey, Nuevo León, Mexico; ^2^Echocardiography Laboratory, Cardiology Service, Hospital Universitario, Universidad Autónoma de Nuevo León, Monterrey, Nuevo León, Mexico

## Abstract

A 48-year-old male with a prior diagnosis of diabetes mellitus presented to the emergency department with malaise and nausea. On work-up, he was found with hyperglycemia and high anion gap metabolic acidosis, with a blood pH < 6.94. A diagnosis of severe diabetic ketoacidosis was established; serum electrolyte analysis showed mild hyperkalemia. On work-up, a 12-lead electrocardiogram was obtained, and it showed an ST-segment elevation on anterior leads that completely resolved with diabetic ketoacidosis treatment. ST-segment elevation myocardial infarction can be a precipitant factor for diabetic ketoacidosis, and evaluation of diabetic patients with suspected myocardial infarction can be challenging since they can present with atypical or little symptoms. Hyperkalemia, which usually accompanies diabetic ketoacidosis, can cause electrocardiographic alterations that are well described, but ST-segment elevation is uncommon. A pseudomyocardial infarction pattern has been described in patients with diabetic ketoacidosis; of note, most of these patients presented severe hyperkalemia. We believe this is of great importance for clinicians because they must be able to recognize those patients that present with electrocardiographic abnormalities secondary to the metabolic alterations and those that can be experiencing actual ongoing ischemia, in order to establish an appropriate and prompt treatment.

## 1. Introduction

Heart tissue is particularly prone to the effects of systemic acidosis and hyperkalemia [1, 2]. Acidosis decreases myocardial contractibility by affecting the excitation-contraction coupling [1], and hyperkalemia causes depolarization of the cardiac-cell resting membrane potential, shortening of the action potential duration, and alterations in the conduction velocity [2]. Diabetic ketoacidosis (DKA) is considered one of the most serious acute complications of diabetes mellitus; it is characterized by hyperglycemia, metabolic acidosis, and increased total body ketone concentrations [3]. Despite volume depletion seen in DKA secondary to vomiting and reduced oral intake, serum potassium levels are typically high at presentation; this is because lack of insulin and the presence of acidosis cause a shift of potassium from the intracellular space to extracellular space which usually resolve with DKA treatment [3, 4]. Thus, as a consequence of the acid-base and potassium derangements, patients with DKA can present electrocardiographic alterations than can be transient and resolve with treatment.

## 2. Case Report

**Figure 1:**
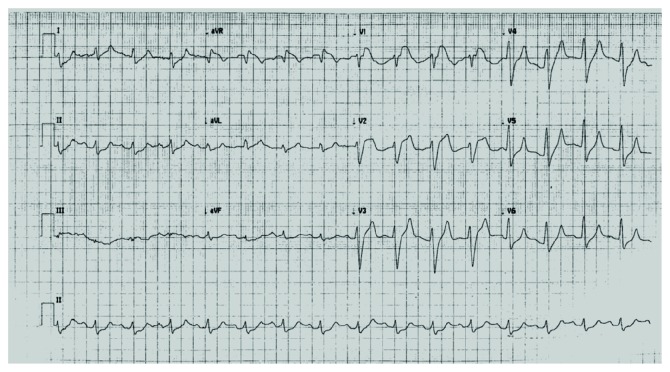
A twelve-lead electrocardiogram showing ST-segment elevation of 4 millimeters in leads V1 and V2, 1 millimeter in aVR, and QRS complex widening, which raised the suspicion of a possible acute coronary syndrome.

**Figure 2:**
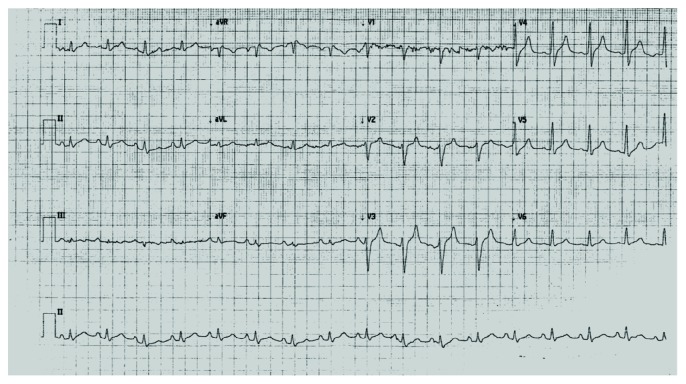
A twelve-lead electrocardiogram performed 1 hour and thirty minutes after initiation of DKA treatment showing complete resolution of ST-segment abnormalities.

A 48-year-old male presented to the emergency department with complaints of malaise and nausea. The past medical record was relevant for type 2 diabetes mellitus that was treated at the time with neutral protamine Hagedorn insulin. On initial examination, he had signs of severe dehydration and was polypneic; blood pressure was 80/60 mmHg, and heart rate was 125 bpm, with an oxygen saturation of 95% at room air. The capillary glucose level was 620 mg/dL; venous blood gases showed a metabolic acidosis with pH < 6.94 and HCO_3_^−^ of 4.1 mEq/L. A severe DKA was diagnosed, and treatment was started with aggressive hydration and IV insulin. Initial electrolytes were Na^+^ 119.6 mEq/L, Cl^−^ 95 mEq/L, and K^+^ 5.7 mEq/L, and serum creatinine was 2.6 mg/dL; the anion gap was high, with 21 mmol/L. A 12-lead electrocardiogram (ECG) was performed and showed an ST-segment elevation of 4 millimeters in V1 and V2 leads and 1 millimeter elevation in aVR lead; also, there was a QRS complex widening and tall T-waves in V3-V5 (Figure 1). Concerns were raised of possible myocardial infarction as the precipitant factor for the DKA. A cardiology consult was required, and a point-of-care cardiac ultrasound was performed, which showed a normal left ventricular ejection fraction and no regional wall motion abnormalities, cardiac troponins where ordered and reported within the normal range. The patient continued on management. After 1 hour and 30 minutes of treatment for DKA, a subsequent ECG was performed, and it showed a complete resolution of the ST-segment elevation (Figure 2). DKA was resolved, and the patient was admitted for monitoring, with a favorable evolution.

## 3. Discussion

Myocardial infarction is a known precipitant factor for DKA; it is responsible of about 1% of the cases, and along with congestive heart failure, it accounts for 28% of deaths in DKA [5]. According to clinical guideliness for diagnosis and treatment of myocardial infarction, ST-segment elevation is considered suggestive of acute coronary obstruction if present in at least two contiguous leads (≥2.5 mm in men < 40 years, ≥2 mm inmen ≥ 40 years, or ≥1.5 mm in women in leads V2-V3) and/or ≥1 mm in any other lead [6]. In the proper clinical context, patients with ST-segment elevation are candidates for reperfusion therapy, which must be instituted promptly since delay in treatment is associated with worst prognosis [6]. Evaluation of suspected myocardial infarction can be particularly challenging in diabetic patients, since they are more likely to present with atypical or little symptomatology [7].

Electrocardiographic alterations related to hyperkalemia are well described [8]. Their presence and presentation depend on the potassium level and also on the rate of raise, acid-base status, other electrolyte disturbances, and medications [8, 9]. Tall, tent-shaped T-waves are the earliest manifestations with mild hyperkalemia (5.5–6.5 mEq/L). With moderate hyperkalemia (6.5–8 mEq/L), a prolonged PR interval, decreased P-wave amplitude, and widening of the QRS complex can be seen. With severe hyperkalemia (>8 mEq/L) there is absence of P-wave, intra- and interventricular conduction blocks, progressive widening of the QRS complex and eventually ventricular fibrillation and asystole. A pseudomyocardial infarction pattern has been described in patients with DKA and hyperkalemia [9–13]. Bellazzini and Meyer reviewed 24 cases of pseudomyocardial infarction in DKA with hyperkalemia reported in the literature [9]. They found that an anteroseptal pattern is the most common presentation (79.2%), followed by a combination of anteroseptal and inferior distribution (12.5%). Of note, 23 of the 24 cases presented with a mean potassium concentration of 8.1 mEq/L. In all these cases, there was no echocardiographic or angiographic finding suggestive of real myocardial ischemia, and there was a complete resolution of electrocardiographic abnormalities after metabolic improvement. There is only one case described in the literature that presented with a pseudomyocardial infarction pattern and normal potassium levels [10]. According to our literature review, no cases of a pseudomyocardial infarction pattern in DKA with mild hyperkalemia have been reported. Possible explanations for the presence of ST-segment elevation in the absence of severe hyperkalemia are the concomitant acid-base disturbances present in our patient, other electrolyte alterations, and possibly a slow coronary blood flow secondary to elevated blood viscosity as a consequence of hyperglycemia [14].

## 4. Conclusion

We present an uncommon electrocardiographic alteration in a patient with severe DKA. Myocardial infarction should always be considered as a possible precipitant of DKA in diabetic patients, but clinicians must be able to recognize when electrocardiographic abnormalities suggestive of myocardial ischemia are related to metabolic derangements in order to avoid unnecessary diagnostic procedures and treatments and also to promptly recognize those patients with actual ongoing ischemia that may benefit from a coronary percutaneous intervention or IV thrombolysis. Normal levels of myocardial necrosis biomarkers, the absence of echocardiographic and angiographic alterations suggestive of myocardial ischemia, and resolution of electrocardiographic alterations with the metabolic control can be reassuring, but close monitoring of the patient during treatment and after resolution is crucial.
